# Investigating the acute cognitive effects of dietary compounds using fNIRS: methodological limitations and perspectives for research targeting healthy adults

**DOI:** 10.3389/fnhum.2024.1493880

**Published:** 2024-12-04

**Authors:** Sélima Zahar, Dimitri Van de Ville, Julie Hudry

**Affiliations:** ^1^Mood and Performance Group, Department of Brain Health, Nestlé Research, Nestlé Institute of Health Sciences, Lausanne, Switzerland; ^2^Medical Image Processing Laboratory, School of Engineering, Ecole Polytechnique Fédérale de Lausanne, Neuro-X Institute, Geneva, Switzerland; ^3^Department of Radiology and Medical Informatics, Faculty of Medicine, University of Geneva, Geneva, Switzerland

**Keywords:** fNIRS, cognition, nutrition, intervention studies, experimental design, processing, neuroimaging, bioactive

## Abstract

The brain’s response to cognitive demands hinges on sufficient blood flow, with changes in brain hemodynamics serving as a reflection of this process. Certain bioactive compounds found in our diet, such as caffeine, polyphenols, and nitrate, can acutely impact brain hemodynamics through diverse neural, vasoactive, and metabolic mechanisms. Functional Near-Infrared Spectroscopy (fNIRS) offers a non-invasive and real-time method to investigate these effects. Despite their potential, fNIRS studies investigating the acute impacts of bioactive compounds on cognition face methodological gaps, especially in controlling confounding factors. Given the impact of these confounding effects, which can be significant due to the relatively limited sample size of such studies, there is a need to refine the methodologies employed. This review proposes recommendations to enhance current methodologies in the research field, focusing on key aspects of the data collection phase, including research design, experimental paradigms, and participant demographics, and their integration into the analysis phase. Ultimately, it seeks to advance our understanding of the effects of bioactive compounds on cognitive functions to contribute to the development of targeted nutritional interventions for improved brain health.

## Introduction

Healthy brain function requires metabolites, glucose, and oxygen to be adequately supplied to neuronal cells through blood flow (Phillips et al., 2016). The response to specific cognitive demands is characterized by the hemodynamic response and it involves an increase in total hemoglobin (HbT) of which an increase in oxygenated hemoglobin (HbO) and a decrease in deoxygenated hemoglobin (HbR) (Jöbsis, 1977).

Certain bioactive compounds from our diet may acutely impact the brain’s response to cognitive demands by stimulating neural, vasoactive, or metabolic mechanisms in the brain. Caffeine, for instance, would acutely enhance cerebral excitability and cognitive responsiveness via adenosine $A_{1}$ receptor and dopamine system activation (Ferré, 2008) while concurrently inducing vasoconstriction in cerebral vessels through interactions with adenosine $A_{2A}$ and $A_{2B}$ receptors (Pelligrino et al., 2010). On the other hand, polyphenols and nitrate have been shown to acutely induce vasodilation in the brain by serving as donors of nitric oxide, a compound known for its ability to widen blood vessels (Sokolov et al., 2013; Hobbs et al., 2013). These compounds can acutely support the metabolic activity in the brain by enhancing mitochondrial efficiency (Lagouge et al., 2006; Baur et al., 2006; Larsen et al., 2011).

Functional Near-Infrared Spectroscopy (fNIRS) provides a non-invasive and real-time approach to investigate the acute impact of bioactive compounds on cortical hemodynamic responses to cognitive stimulation (Dodd et al., 2015; Kennedy and Haskell, 2011; Wightman et al., 2015; Wightman et al., 2014; Wightman et al., 2012; Kennedy et al., 2010; Heilbronner et al., 2015; Higashi et al., 2004; Yuan et al., 2020). This technique employs scalp optical probes which integrate both detectors and emitters, forming pairs or channels for measurements of [HbO] and [HbR] changes within the cortex. Specifically, the detector measures the amount of emitted light that is absorbed by HbO and HbR, as chromophores. These variations in light intensity are then converted into changes in the concentration of [HbO] and [HbR] using the modified Beer–Lambert law, which establishes a relationship between light absorption, path length and chromophore concentration (Scholkmann et al., 2014). The unique capability of fNIRS to simultaneously measure changes in both [HbO] and [HbR] sets it apart from functional magnetic resonance imaging (fMRI), which typically provides a signal based on the relative difference between HbO and HbR levels (Chen et al., 2020). Additionally, fNIRS is effective at detecting activation in superficial cortical regions linked to executive cognitive functions which can be visualized through functional activation maps. It is important to note that the penetration depth (1–2 cm) and the spatial resolution (2–4 cm) of these maps are lower compared to what is obtained with fMRI (Chen et al., 2020; Joanette et al., 2008). fNIRS offers slightly better temporal resolution than fMRI, achieving approximately 0.1-s precision. However, fNIRS does not match the high temporal precision of electroencephalography (EEG) (Chen et al., 2020). What distinguishes fNIRS from other techniques such as fMRI and EEG is its user-friendly nature and portability. These features make fNIRS particularly suitable for acute studies aimed at short-term investigations, where deploying more complex and resource-intensive techniques can be challenging (Pinti et al., 2018).

Despite the potential of fNIRS in the field, there are significant gaps in the methodological approach used for its application. Studies exploring the acute effects of caffeine, polyphenols, or nitrate on cortical hemodynamics have yielded inconsistent findings, displaying variability within their own data or in comparison to other studies evaluating the same component during similar cognitive testing (Kennedy and Haskell, 2011; Wightman et al., 2012; Kennedy et al., 2010; Heilbronner et al., 2015; Yuan et al., 2020; Wightman et al., 2015; Best et al., 2019). These inconsistencies may stem from confounding factors, including individual differences (Beishon et al., 2021) and measurement bias (Ishii et al., 2014). The prevalence of these confounding factors, often unaccounted for during both the data collection and analysis stages underscores the need for methodological refinement. This is particularly important considering the relatively small sample size and targeted effect size in the field, which increase susceptibility to confounding effects (Smeets et al., 2019). Consequently, there is a need to enhance the methodologies used to ensure more consistent and reliable outcomes in the research field.

In this paper, we present a methodological review centered on studies using fNIRS to investigate the acute effects of dietary bioactive compounds on cortical hemodynamics during cognitive tasks, specifically in healthy adults. Our approach is three-fold. First, we summarize the existing literature, identifying discrepancies in studies involving established bioactive pathways and expected outcomes. Second, we examine how these discrepancies may stem from confounding factors related to methodological limitations in data collection and analysis. Finally, we put forth recommendations to enhance current methodologies in the field, aiming to help practitioners optimally address key aspects of the data collection and incorporate them into the analysis phase.

## Review of evidence

In this section, we detail our review search strategy and the search terms employed, followed by a summary of the evidence obtained, which is also presented in Table 1.

**Table 1:** Summary of studies assessing the acute effects of dietary components on cortical hemodynamics during cognitive testing using fNIRS.

Interventions	Design	Population	Acquisition set-up	Experimental paradigm	Preprocessing and analysis	Results	References
75 mg caffeine, 75 mg caffeine +50 mg L-theanine and 50 mg L-theanine in a capsule	Placebo-controlled, double-blind, randomized, counterbalanced and cross-over 4 arms, 1 visit per arm (pretest and posttest)	24 healthy adults (14 females, mean age: 21.8 ± 3.19 SD yo) with a stratification by caffeine consumption status	Stationary system, probing of PFC area with 2 channels	Battery of SS, SRT, RVIP, RT and Stroop tasks	Baseline correction Mean-based analysis using ANOVA	Caffeine vs. placebo 0–30 min: Lower [HbO] & higher [HbR] in non-habitual consumers 30–80 min: Lower [HbO] and higher [HbR], more pronounced effect in non-habitual consumers Caffeine + L-theanine vs. placebo 0–30 min: Higher [HbO] 30–80 min: Higher [HbR] L-theanine: No change observed	Dodd et al. (2015)
75 mg caffeine in a capsule	Placebo-controlled, double-blind, randomized, counterbalanced and cross-over 2 arms, 1 visit per arm (pretest and posttest)	20 healthy young adults (10 females, age range 19–28 yo) with a stratification by caffeine consumption status	Stationary system, probing of PFC area with 2 channels	Battery of SS and RVIP tasks	Baseline correction Mean-based analysis using ANOVA	Caffeine vs. placebo 40–70 min: Lower [HbT], more pronounced effect in non-habitual consumers	Kennedy and Haskell (2011)
200 mg caffeine in a tablet	Placebo-controlled, sequential 1 visit for 2 arms	10 healthy adults (7 females, age: 29.1 ± 1.6 SEM yo) comprising only of moderate caffeine consumers	Stationary system, probing of IFC area with 52 channels	Verbal n-back task (*n* = 0.2)	Baseline correction & band-pass filtering (0.001–0.5 Hz) Mean-based analysis using non-parametric paired Wilcoxon signed rank tests	Caffeine vs. placebo 30 min: Respectively lower and higher [HbO] and [HbR] in the left hemisphere & lower [HbO] in the right hemisphere	Heilbronner et al. (2015)
A 3 mg/kg dose of caffeine incorporated in a 210 mL coffee cup	Placebo-controlled, cross-over 1 visit (pretest and posttest) for 2 arms	31 healthy adults (16 females, age range: 21–24 yo)	Stationary system, probing of PFC area with 20 channels	Modified color-word matching Stroop task	Band-pass filtering (0.01–0.2 Hz) & removal of channel below/above a light intensity threshold Mean-based analysis using ANOVA	Caffeine vs. placebo 0–20 min: Higher [HbO] in the left dorsolateral hemisphere and right ventrolateral hemisphere under rest conditions 20 min to the end: Higher [HbO] in the dorsolateral and left ventrolateral hemispheres	Yuan et al. (2020)
250 or 500 mg of trans-resveratrol in capsules	Placebo-controlled, double-blind, randomized, counterbalanced, cross-over 1 visit (pretest and posttest) per arm	24 healthy adults (20 females, age range 18–25 yo)	Stationary system, probing of PFC area with 2 channels	Battery of SS, RVIP and n-back tasks	Baseline correction Mean-based analysis using ANOVA	250 mg and 500 mg trans-resveratrol vs. placebo: 0–45 min: Higher [HbR] 45–80 min: Higher [HbO] and [HbT]	Kennedy et al. (2010)
250 mg resveratrol alone or in combination with 20 mg piperine in capsules	Placebo-controlled, double-blind, randomized, counterbalanced and cross-over 1 visit (pretest and posttest) per arm	23 (19 females, mean age 21 +/− 3.2 SD yo)	Stationary system, probing of PFC area with 2 channels	Battery of SS, RVIP and n-back tasks	Baseline correction Mean-based analysis using ANOVA	No difference observed	Wightman et al. (2014)
500 mg resveratrol in a capsule	Placebo controlled, double-blind, randomized, parallel design 1 visit (pretest and posttest)	60 healthy adults (39 females, age range 18–29 yo)	Stationary system, probing of PFC area with 2 channels	Battery of SS, RVIP and n-back tasks	Baseline correction Mean-based analysis using ANOVA	500 mg resveratrol vs. placebo: 40–80 min: Higher [HbT] and [HbO] & lower [HbR] under absorption (~0–40 min)	Wightman et al. (2015)
135 or 270 mg EGCG in a capsule	Placebo-controlled, double-blind, randomized, counterbalanced and cross-over 1 visit (pretest and posttest) per arm	27 healthy adults (16 females, age range 18–30 yo)	Stationary system, probing of frontal cortex area with 2 channels	Battery of SS, odd-ball, RVIP, Stroop or SRT tasks	Baseline correction Mean-based analysis using ANOVA	135 mg EGCG vs. placebo: 45–90 min: Lower [HbO] and [HbT]	Wightman et al. (2012)
450 mL juice with 5.5 mmol of nitrate (drink)	Placebo-controlled, double-blind, parallel design 2 arms, 1 visit per arm (pretest and posttest)	40 healthy young adults (28 females, age range 18–27 yo)	Stationary system, probing of frontal cortex area with 2 channels	Battery of serial subtraction and RVIP tasks	Baseline correction Mean-based analysis using ANOVA	Nitrate vs. placebo: 90-140 min: Modulation of [HbT]	Wightman et al. (2015)
60 mL of Montmorency Tart cherry concentrate (drink)	Placebo-controlled, double-blind, randomized, counterbalanced and cross-over 2 arms, 1 visit per arm (pretest and posttest)	30 healthy middle-aged adults (10 females, 50 +/− 6 SD yo)	Stationary system, probing of PFC area with 20 channels	Battery of digit vigilance, RVIP and Stroop tasks	Baseline correction Mean-based analysis using ANOVA	Montmorency cherry tart vs. placebo: 1 h: Higher [HbT] and [HbO]	Keane et al. (2016)
Mix of 300 mg *Bacopa Monnieri*, 100 mg American ginseng Panax quinquefolieus (inc. 9 mg of ginsenosides) and 100 mg whole coffee fruit (inc. 40 mg of chlorogenic acid) incorporated in tablets	Placebo-controlled, double-blind, parallel design 2 arms, 1 visit per arm (pretest and posttest)	40 healthy adults (21 females, 34.46 ± SD 12.95yo)	Stationary system, probing of PFC area with 8 channels	Stroop and n-back (*n* = 1,2, and 3) tasks	Removal of channel below signal-to-noise ratio threshold, motion artifact correction using PCA, band-pass filtering (0.01–0.5 Hz) & baseline correction Mean-based analysis using ANOVA	Mix vs. placebo: > 45 min: Lower [HbO] during the 3-back & Stroop tasks	Best et al. (2019)

ANOVA, Analysis of Variance; ECCG, Epigallocatechin gallate; HbO, Oxygenated hemoglobin; HbR, Deoxygenated hemoglobin; HbT, Total hemoglobin; PCA, Principal Component Analysis; PFC, Prefrontal Cortex; Pretest and posttest, Pre- and pro-intervention testing, respectively; RT, Reaction Time; RVIP, Rapid Visual Information Processing; SD, Standard deviation; SEM, Standard error of the mean; SRT, Simple Reaction Time; SS, Serial Subtraction.

## Literature search

Our methodological review focuses on studies using fNIRS to investigate the acute—within a single day—effects of dietary bioactive compounds on cortical hemodynamics during cognitive tasks, specifically in healthy adults aged 18–59 years. The review specifically considers both isolated and combined bioactive compounds that involve established pathways.

The corpus of reviewed studies was obtained through a comprehensive literature search performed in the Medline database, employing specific search terms including (“fNIRS” OR “cortical hemodynamic” OR “cerebral hemodynamic” OR “cerebral blood flow”) AND (“acute”) AND (“nutrition” OR “dietary” OR “bioactive” OR “mix” OR “polyphenol” OR “flavonoid” OR “resveratrol” OR “caffeine” OR “nitrate”) AND (“cognition” OR “cognitive” OR “cognitive executive function” OR “cognitive performance” OR “working memory” OR “cognitive inhibition” OR “behavior”).

From the conducted literature search, we exclusively selected the studies that provided comprehensive information on research design, experimental paradigms, participant characteristics, as well as analysis techniques. Further, only original research articles written in the English language were considered.

Ultimately, the corpus consisted of 11 articles, comprising four on caffeine, five on polyphenols, and one on nitrate, either in isolation or combined with other components (Table 1).

## Summary of evidence

Evidence from various studies demonstrates the intricate influence of bioactive compounds on the brain during its activation by cognitive tasks (Table 1). Research shows evidence of polyphenols increasing cortical hemodynamics during cognitive executive functioning tasks such as the Rapid Visual Information Processing (RVIP) task (Kennedy et al., 2010; Wightman et al., 2015; Keane et al., 2016). Conversely, multiple investigations highlight that caffeine consistently reduces the hemodynamic response in the frontal cortex during cognitive executive function tasks such as the n-back or Stroop tasks (Dodd et al., 2015; Kennedy and Haskell, 2011; Heilbronner et al., 2015). Notably, the combination of L-Theanine with caffeine has been observed to counteract this effect (Dodd et al., 2015; Rogers et al., 2008).

The acute testing of dietary bioactive compounds on cognitive-related cortical hemodynamics has also generated inconsistent results (Table 1). For instance, studies on the effects of resveratrol, despite using similar doses and cognitive tasks, have reported contradictory outcomes—some indicating an increase in cortical hemodynamics (Kennedy et al., 2010) and others show no change (Wightman et al., 2014). Disparities among channels within the same cortical region, particularly the right ventrolateral prefrontal cortex (PFC), have been noted in the caffeine study conducted by Yuan et al. (2020). Other caffeine interventions revealed differences among individuals. For example, in the studies by Kennedy and Haskell (2011) and Dodd et al. (2015), a subgroup of non-habitual caffeine consumers exhibited lower cortical hemodynamic responses compared to habitual consumers. Finally, whether involving polyphenol, nitrate, or caffeine interventions, studies revealed important variation in measurement over time (Kennedy and Haskell, 2011; Wightman et al., 2015; Wightman et al., 2015).

## Limits: Neglected confounding factors

This section examines how individual factors, biases, and artifacts confound fNIRS measurements in the reviewed studies. It is organized according to these confounding factors and discuss their potential to introduce variability in the results, particularly when methodologies fail to account for them.

## Individual differences

Individual differences, such as gender (Cosgrove et al., 2007), age (Beishon et al., 2021), physical fitness (Davenport et al., 2012) and dietary habits (Addicott et al., 2009), can significantly contribute to discrepancies in fNIRS measurements.

The role of gender in cortical hemodynamics has been highlighted in existing research. For example, men would metabolize caffeine at a higher rate than women, leading to variations in caffeine metabolites and different magnitudes of induced effects (Rasmussen et al., 2002). Additionally, gender differences can directly impact the hormonal and neurotransmitter regulations involved in cognitive functions (Cosgrove et al., 2007). However, some studies exhibited an overrepresentation of females compared to males (Wightman et al., 2014; Kennedy et al., 2010), potentially causing an uneven distribution of individual factors within each intervention arm, particularly in parallel design studies (Wightman et al., 2015).

Age represents another noteworthy factor. It is associated with changes in compensatory mechanisms that are activated to cope with cognitive demand and these changes can be observed in neural patterns (Beishon et al., 2021). Age-related variations could also link to poorer endothelial functions, potentially enhancing the efficacy of components such as polyphenols in older subjects (Lamport et al., 2015). Studies that do not account for the age range within a population—where ages can span from 21 to 50 years (Higashi et al., 2004)—may face challenges with age acting as a confounding factor.

Dietary habits, particularly chronic caffeine consumption, can influence the results of studies in the field, particularly those focusing on caffeine. Indeed, chronic caffeine consumption can upregulate vascular adenosine receptors such as those responsible for vasoconstriction (Varani et al., 1999). While some studies have accounted for caffeine consumption level by stratifying participants based on this factor or by including it as a covariate in the analysis model (Dodd et al., 2015; Kennedy and Haskell, 2011; Wightman et al., 2015; Wightman et al., 2014; Wightman et al., 2012; Kennedy et al., 2010; Heilbronner et al., 2015), others neglected the potential impact of caffeine consumption level (Higashi et al., 2004; Yuan et al., 2020).

Finally, other factors include physical activity level or cognitive ability. Physical activity level can directly impact the reserve of cortical tissue oxygenation (Davenport et al., 2012). Furthermore, cognitive ability has been hypothesized to correlate with regional metabolic rates in the brain. According to the neural efficiency hypothesis, this relationship is often assumed to be negative, suggesting that individuals with higher cognitive ability may exhibit lower metabolic rates in certain brain regions while performing cognitive tasks. However, it is important to note that this relationship can be influenced by other factors, e.g., task complexity (Dunst et al., 2014).

## Biases

Several biases may contribute to discrepancies in the reviewed fNIRS studies, including selection or observation bias as well as carry-over effects.

Selection and observation biases arise from investigator’s scientific beliefs, along with the cultural and social influences conveyed by participants (Khadilkar and Khadilkar, 2020; Meuli and Dick, 2018). Selection bias might occur when participants are consistently chosen from the same pool or when the allocation to an intervention is made at the investigator’s discretion without randomization (Meuli and Dick, 2018). Since many studies in this review originate from the same research group, with, potentially, similar decision factors among investigators, the impact of this bias on reproducibility might have been mitigated (Dodd et al., 2015; Kennedy and Haskell, 2011; Wightman et al., 2014; Wightman et al., 2012; Kennedy et al., 2010; Wightman et al., 2015).

Other biases can manifest into systematic errors in data collection, interpretation, or reporting due to the decisions made by investigators and are called “observation bias.” In some of the reviewed studies, an observation bias may have been introduced through the investigator’s decision to analyze changes in either [HbO] or [HbR] (Kennedy and Haskell, 2011; Yuan et al., 2020). Furthermore, certain post-hoc analyses conducted in the reviewed studies (Wightman et al., 2015) are prone to observation bias, as they may have been influenced by changing beliefs or perspectives following initial observations (Meuli and Dick, 2018). Such bias can be due to the absence of procedures to mask or blind the allocation of interventions to participants (Heilbronner et al., 2015; Yuan et al., 2020).

In addition to these biases, Time-on-Task (ToT) and carry-over effects can introduce systematic errors in data collection, resulting in discrepancies in fNIRS measurements.

The ToT effect arises when prolonged engagement in cognitive tasks induces mental fatigue, ultimately resulting in a decline in cognitive performance. As participants engage in tasks for extended periods, they may experience increased mental fatigue, prompting the brain to develop coping strategies, particularly within the frontal regions. However, when cognitive demands become excessive, the brain’s ability to recruit cortical resources may be inhibited, which is reflected in fNIRS measurements (Ishii et al., 2014; Sturm and Willmes, 2001). Certain studies with prolonged experimental sessions, such as those involving intervention visits on the same day (Heilbronner et al., 2015) or a battery-of-task paradigm (Dodd et al., 2015; Kennedy and Haskell, 2011; Wightman et al., 2015; Wightman et al., 2012; Kennedy et al., 2010; Wightman et al., 2015), might have triggered a ToT effect during data collection. Specifically, two of the caffeine intervention studies reviewed here, where the placebo and caffeine products were administered sequentially within the same experimental session, could potentially be susceptible to a ToT effect (Heilbronner et al., 2015; Yuan et al., 2020). A ToT effect was notably suggested by an increase in subjective mental fatigue across tasks in one of the studies reviewed (Wightman et al., 2014). Nevertheless, most studies using a battery-of-task paradigm (Dodd et al., 2015; Kennedy and Haskell, 2011; Wightman et al., 2014; Wightman et al., 2012; Kennedy et al., 2010; Heilbronner et al., 2015; Wightman et al., 2015) considered the ToT effect in their analysis.

The carry-over effect—referring to the influence of a prior cognitive task or trial on subsequent ones—also plays a significant role in shaping cognitive-evoked hemodynamics. This effect may engage some brain regions, e.g., the PFC, to resolve conflicting information and guide memory retrieval, thereby influencing their activation during task performance (Preston and Eichenbaum, 2013). The carry-over effect can be triggered by tasks employing a rapid event-related design owing to the closely spaced stimuli (100/min), leading to higher influence of prior stimuli on subsequent ones. This type of design has been frequently used in the reviewed studies (Dodd et al., 2015; Kennedy and Haskell, 2011; Wightman et al., 2015; Wightman et al., 2014; Wightman et al., 2012; Kennedy et al., 2010; Wightman et al., 2015; Keane et al., 2016). Nonetheless, these studies neglected to incorporate experimental strategies aimed at mitigating this effect.

## Motion and physiological artifacts

Variation in measurements within the reviewed studies may also arise from motion and physiological artifacts.

Motion artifacts, whether task-evoked or spontaneous, arise from probe displacement or oscillation due to imperfect contact with the scalp, leading to rapid signal changes or spikes that significantly distort the fNIRS signal and induce spurious cognitive-evoked responses (Dans et al., 2021; Zhang et al., 2023).

In the reviewed studies, techniques to handle motion were lacking. Specifically, the analysis of either [HbO] or [HbR] in many studies (Higashi et al., 2004; Yuan et al., 2020) may have hindered researchers’ ability to distinguish a motion artifact from a cognitively-evoked hemodynamic response, typically identifiable by a pattern of positively correlated changes in [HbO] and [HbR] (Tachtsidis and Scholkmann, 2016). When motion artifacts were detected, they were corrected in a conservative manner, often relying on thresholding techniques based on data quality metrics like channel voltage, as applied in Yuan et al. (2020). However, the important loss of trials resulting from such methods is particularly concerning in experiments with a limited number of trials, such as acute experiments (Brigadoi et al., 2014). Moreover, the studies predominantly employed averaging techniques (Dodd et al., 2015; Kennedy and Haskell, 2011; Wightman et al., 2015; Wightman et al., 2014; Wightman et al., 2012; Kennedy et al., 2010; Heilbronner et al., 2015; Higashi et al., 2004; Yuan et al., 2020), which might not adequately tackle the non-identically distributed motion artifacts caused by probe displacement or oscillation on the scalp over time (Huppert, 2016).

Physiological artifacts arise from rhythmic fluctuations in both cerebral and extra-cerebral vessels that are illuminated by NIRS light, including those in the scalp, skull, and surrounding tissues. In cerebral vessels, fluctuations associated with heart rate (~1 Hz) and respiration rate (~0.3 Hz) occur. These fluctuations are regulated by the cardiac, autonomic nervous, and respiratory systems (Willie et al., 2014) and they can also be triggered by experimental procedures. For example, during cognitive tasks, activation of the sympathetic nervous system can reduce blood flow to the scalp and superficial tissues of the head, leading to fluctuations in fNIRS signals. Similarly, gustatory stimulation resulting from the ingestion of dietary components can lead to changes in cortical hemodynamics (Kirilina et al., 2012). Beyond cerebral tissues, extra-cerebral physiological oscillations involve vasomotion waves (~0.02 Hz) (Willie et al., 2014), as well as spontaneous hemodynamic fluctuations (~0.1 Hz) (Julien, 2006).

The reviewed fNIRS studies often lack techniques to handle physiological artifacts. Specifically, studies employing tasks with fixed time intervals between stimuli (Best et al., 2019) may be susceptible to physiological artifacts, as these intervals might unintentionally coincide with physiological oscillations, like respiration (Tachtsidis and Scholkmann, 2016; Yücel et al., 2021). However, physiological artifacts were only partially addressed in the analysis (Dodd et al., 2015; Kennedy and Haskell, 2011; Wightman et al., 2014; Wightman et al., 2012; Kennedy et al., 2010; Wightman et al., 2015). Certain studies have attempted to mitigate these artifacts by employing a zero-order filter, but it may not fully account for the presence of very low-frequency components and gradual trends associated with motion artifacts in the signal (Heilbronner et al., 2015; Yuan et al., 2020; Best et al., 2019). The complexity escalates when attempting to filter out physiological artifacts while keeping the signal of interest. Indeed, physiological artifacts and cognitively-induced hemodynamic responses can operate within close frequency ranges (Pinti et al., 2019). Specifically, in the n-back task of Heilbronner et al. (2015), the cognitive stimulation frequency of 0.4 Hz closely mirrored physiological oscillations, particularly respiration. Therefore, the band-pass filter with cut-off frequencies set at 0.001 and 0.5 Hz may not have effectively eliminated physiological artifacts. Additionally, all the studies used averaging analysis, which entails examining fNIRS segments averaged over the duration of the task, from onset to completion (Dodd et al., 2015; Kennedy and Haskell, 2011; Wightman et al., 2015; Wightman et al., 2014; Wightman et al., 2012; Kennedy et al., 2010; Heilbronner et al., 2015; Higashi et al., 2004; Yuan et al., 2020). This analysis technique assumes that the fNIRS signal follows a Gaussian shape with physiological noise being independent, and identically distributed throughout the experiment. In practice, physiological noise can show variation throughout the experiment due to changes in experimental procedures (Kirilina et al., 2012). Moreover, physiological noise in fNIRS measurements can be interdependent with its own historical components or with other noise sources. This pattern, known as serial correlation, is particularly significant in fNIRS recordings due to the high sampling rate (Huppert, 2016).

## Perspectives for methodological improvements

**Figure 1:**
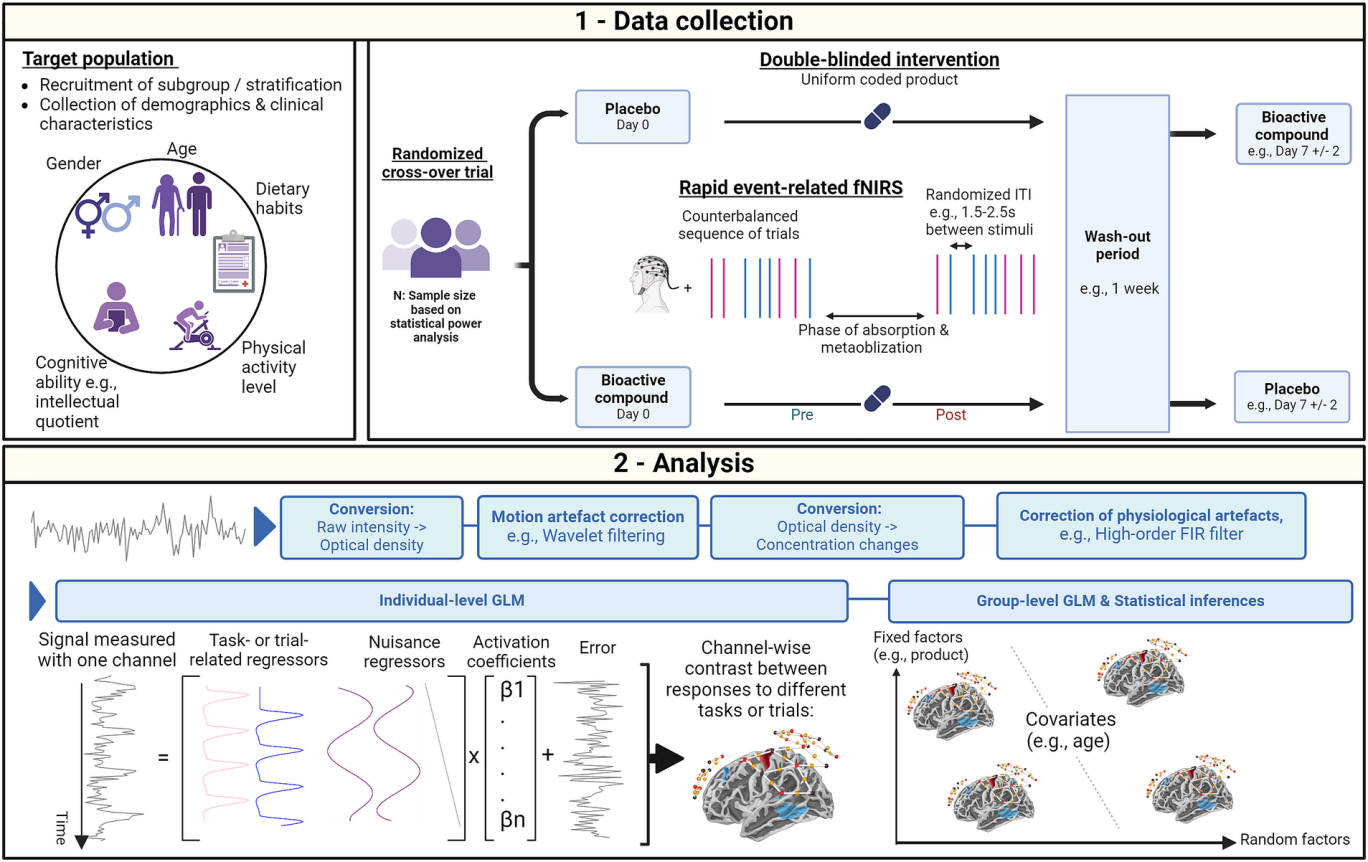
Methodological considerations for functional near infrared spectroscopy (fNIRS) studies investigating the acute cognitive effects of bioactive compounds in healthy adults. (1) During data collection, individual factors can be controlled by recruiting participants from specific subgroups or stratifying the population according to these factors. To further characterize the population, individual factors can be collected and used as covariates in the analysis model. To ensure precise data collection, the study design needs to be optimized, which involves unbiased participant allocation strategies through randomization and masked interventions. Individual variability can further be controlled through a cross-over allocation to interventions. The intervention visits should however be separated by a specific period aimed at mitigating any carry-over effect while allowing a wash-out of the product effect. To generate reliable contrast in the hemodynamic responses to various task- or trial-related conditions, a rapid counter-balanced event-related paradigm can be employed. This paradigm allows optimizing inter-trial intervals (ITI) and randomizing trial presentation probabilities to further enhance the contrast. (2) To preprocess each individual’s fNIRS signal during the analysis stage, techniques such as wavelet filtering for motion artifact correction and high-order Finite Impulse Response (FIR) filtering for physiological noise correction can be used. Individual-level contrasts for each channel or region-of-interest can be extracted using the General Linear Model (GLM). Extending the GLM to the group level allows for the analysis of these contrasts with respect to fixed factors, such as the administered product. The group-level model also allows for the integration of covariates and random factors to address individual variability. This figure was created with BioRender.com.

This section presents methodological considerations to address limitations that contribute to confounding factors in fNIRS measurements, offering recommendations for key aspects of data collection, including research design, experimental paradigms, participant demographics, and analysis (Figure 1).

## Study design and population

To effectively isolate the acute effects of bioactive compounds from individual variability and research bias, it is essential for studies to refine their design and explicitly define their target populations.

Several design considerations can help achieve this.

One effective design is the cross-over allocation, where each participant receives the interventions in successive visits. This approach minimizes individual variability by allowing every participant to serve as their own control. To mitigate potential carry-over effects between visits and ensure an adequate wash-out period for the product, it is advisable to schedule these visits at specific intervals (Lim and In, 2021; Bell et al., 2018).

Employing a double-blind approach is also essential to mitigate research biases. Indeed, researchers must remain unaware of the specific interventions participants receive to avoid selection and observation biases (Meuli and Dick, 2018). Achieving a double-blind protocol involves concealing the intervention assignments from both the researcher and the participant. This can be accomplished by using capsules as a product format, ensuring uniformity in size, color, and odor. However, product uniformization may not be appropriate for interventions that focus on the sensorial aspects of the product. Additionally, product coding helps prevent researchers from identifying which intervention has been assigned to the participant (Monaghan et al., 2021).

Randomization further reduces research biases, especially selection biases. By randomly allocating participants, individual factors that could potentially influence the study outcomes are evenly distributed across the intervention arms (Lim and In, 2019). Additionally, counter-balancing the order of intervention assignment can help prevent the carry-over effect (Brooks, 2012).

In addition to these design considerations, several methods can be employed to explicitly define the target population.

Stratification is one approach that involves categorizing the target population into distinct subgroups or strata based on specific individual factors (Lim and In, 2019). This guards against type I errors that might arise due to imbalanced groups. However, stratification can be challenging in small studies due to limited sample size, potentially leading to underpowered analyses (Kernan et al., 1999).

When stratification proves unfeasible or overly restrictive, especially in small studies, an alternative approach is to recruit volunteers from a single stratum or subgroup. This method, however, compromises the study’s generalizability, thereby limiting the broader applicability of the findings.

## Experimental paradigm

When evaluating the acute effects of bioactive compounds, it is also essential to adequately select the experimental paradigm to control for confounding factors (e.g., carry-over effect) and effectively contrast cortical hemodynamic responses across different cognitive tasks or trial conditions (e.g., congruent versus incongruent trials).

Among the prevalent paradigms in fNIRS research, event-related and block-related paradigms stand out due to their standardized approaches, ensuring consistent approximations of cortical hemodynamic responses to cognitive stimulation (Luke et al., 2020). In an event-related paradigm, tasks are structured into trials with varying ITI, whereas a block-related paradigm organizes tasks into blocks comprising closely spaced and successive similar or dissimilar trials (Josephs and Henson, 1999). To determine the appropriate paradigm for a specific task, consulting the existing literature on task requirements is recommended.

The event-related paradigm, compared to the block-related paradigm, is advantageous in capturing brief cortical activation by smoothly transitioning between different trial types, while also offering flexibility to refine the paradigm and overcome its lower contrast-to-noise ratios (Twamley et al., 2006). These refinements target both the duration of ITI and the presentation order of trials. Specifically, the duration of ITI should not be unduly prolonged to avoid cognitive biases arising from anticipation or boredom. Moreover, implementing an inter-stimulus interval of 2–5 s according to a rapid-related design can increase the number of stimuli presented, intensifying the contrast-to-noise ratio (Buckner et al., 1998). To minimize the interference of physiological artifacts during cognitive stimulation, it is also recommended to randomize the ITI (Tachtsidis and Scholkmann, 2016; Yücel et al., 2021). Employing pseudo-randomization to counterbalance the presentation of trial types can further permit to mitigate biases such as the carry-over effect.

Administering task batteries can further improve the contrast-to-noise ratio, provided they offer flexibility in adjusting administration time to effectively manage the occurrence of the ToT effect (Zygouris and Tsolaki, 2015; Roalf et al., 2014). Finally, the paradigm should enable a pre-post intervention analysis by integrating cognitive-related fNIRS measurements both before and after the intervention. The interval between these measurements should align with the absorption and metabolization processes of the dietary components under study to accurately capture their effects on cortical hemodynamics and cognitive functions (Dimitrov and Rumrill Jr, 2003).

## Pre-processing

Once we have established an adequate experimental paradigm, we can delve into the processing phase to effectively address motion and physiological artifacts from fNIRS measurements.

A typical fNIRS pre-processing pipeline involves converting raw intensity data to optical density, correcting motion artifacts, transforming optical density into concentration changes, and filtering out physiological oscillations (Huppert et al., 2009). Motion artifacts can be mitigated using techniques like principal component analysis (Zhang et al., 2005), spline interpolation (Scholkmann et al., 2010), Kalman filtering (Izzetoglu et al., 2010), wavelet filtering (Molavi and Dumont, 2012), and correlation-based signal improvement (Cui et al., 2010). Wavelet filtering stands out as one of the most sensitive approaches for rectifying motion artifacts. Its efficacy lies in its ability to discern signal components affected by motion, spanning from spikes, baseline shifts and low-frequency variations. However, the successful implementation of wavelet filtering relies on precisely tuning its parameters, especially the probability threshold for filtering signal components as motion artifacts (Brigadoi et al., 2014; Molavi and Dumont, 2012; Cooper et al., 2012). The next step in the pipeline involves filtering out physiological oscillations. One common approach is to use a band-pass filter to effectively remove these oscillations. However, caution must be taken to avoid compromising the signal-to-noise ratio by eliminating frequencies linked to the hemodynamic response. Simulation results by Pinti et al. (2019) especially recommend setting a lower cut-off at 0.01 Hz, which would allow to eliminate low-frequency physiological oscillations, while the higher cut-off should exceed the stimulation frequency. They suggest using a finite impulse response (FIR) filter due to its stability and robustness against signal distortion, compared to other common filters such as the infinite impulse response (IIR) filter. In cases where physiological oscillations cannot be removed without affecting the frequential component related to the cognitive stimulation, alternative approaches such as adaptive filtering can be explored. Adaptive filtering selectively removes physiological components prominent in extracerebral tissues, which are measured by auxiliary channels like short-separation channels (Zhang et al., 2007). These channels must be properly coupled to the scalp to avoid inflating motion artifacts during signal pre-processing (Yücel et al., 2021).

## Statistical modeling

After correcting the fNIRS measurements for artifacts, statistical modeling can be employed to estimate cognitively evoked changes.

Evoked changes in hemodynamics are estimated from variations in [HbO] and [HbR] for each individual and each fNIRS channel. Traditionally, studies have estimated these changes by averaging channel data across tasks or trials, followed by aggregating the data from channels into a region-of-interest (ROI) (Dodd et al., 2015; Kennedy and Haskell, 2011; Wightman et al., 2015; Wightman et al., 2014; Wightman et al., 2012; Kennedy et al., 2010; Heilbronner et al., 2015; Higashi et al., 2004; Yuan et al., 2020). Using a General Linear Model (GLM) approach significantly improves the estimation of task-evoked cortical activity compared to simple averaging methods. The GLM allows for the integration of task- or trial-related regressors as well as regressors accounting for motion and physiological artifacts (Huppert, 2016).

At the group-level, the GLM facilitates the analysis of the effects of acute nutrition interventions on fNIRS data under specific experimental conditions. This model incorporates regressors for fixed factors such as the administered product and/or experimental condition, while accommodating covariates and random factors to address individual variability (Huppert, 2016; Tak and Ye, 2014). Consequently, contrasts (e.g., comparing cortical hemodynamic responses following caffeine intake versus a placebo) can be estimated using the relevant fixed factors (e.g., product) (Smeets et al., 2019).

## Discussion

This paper offers a comprehensive review of methodologies employed in using fNIRS to evaluate the acute impact of bioactive compounds on cortical hemodynamics during cognitive testing in healthy adults (Dodd et al., 2015; Kennedy and Haskell, 2011; Wightman et al., 2014; Wightman et al., 2012; Wightman et al., 2015; Best et al., 2019; Keane et al., 2016; Wightman et al., 2018). While acknowledging the potential advantages for fNIRS application in the field, our findings highlight the need to refine methods to address confounding factors, including gender (Cosgrove et al., 2007), ToT effect (Ishii et al., 2014) or researcher bias (Khadilkar and Khadilkar, 2020).

In refining methodologies, we propose more robust research design, experimental paradigm, and analysis techniques. Optimization of study design involves implementing randomization and masking for participant allocation while controlling for individual variability using cross-over designs and stratified sampling (Lim and In, 2019; Brooks, 2012; Kernan et al., 1999). In addition, we highly recommend the use of experimental paradigms such as the rapid counter-balanced event-related paradigm as it allows to effectively extract cortical hemodynamic responses that exhibit significant contrasts across different task or trial conditions (Buckner et al., 1998). To pre-process the data collected and address motion artifacts, we suggested approaches such as principal component analysis (Scholkmann et al., 2010) and wavelet filtering (Brigadoi et al., 2014; Molavi and Dumont, 2012; Cooper et al., 2012) which can be complemented by higher-order FIR filter or adaptive filtering techniques to correct for physiological artifacts (Zhang et al., 2007). In the processing phase, employing the GLM to estimate, for each individual, the cortical hemodynamic responses induced by different task or trial conditions is suggested for its capability to enhance depth and precision of estimates compared to other methods such as averaging analysis (Huppert, 2016). This model can be extended to the group level to assess the impact of fixed factors (e.g., product or experimental condition). It also allows to account for individual variability by incorporating covariates or random factors. Furthermore, a predefined statistical plan is essential for ensuring a well-powered study and minimizing potential researcher bias (Khadilkar and Khadilkar, 2020).

To elevate the application of fNIRS in the field, further advancements are needed. Among these advancements, a key focus should be on the integration of a hardware system designed for motion correction (Pinti et al., 2018; Pinti et al., 2020). We also need to better understand the mechanisms underlying cognitive processes and how they can be modulated by bioactive compounds. Exploring beyond the frontal region holds promise for achieving such understanding. Indeed, measuring regional effects becomes essential when studying substances like caffeine, which exhibit uneven distribution of receptors across the cortex (Pelligrino et al., 2010). Expanding research to encompass areas like the parietal region, which has been identified as activated by caffeine during cognitive executive function tasks, is thus essential (Nowrangi et al., 2014; Becker et al., 2022; Schmidt et al., 2014). Moreover, closing the gap between preclinical and clinical evidence is key. Such gaps exists for compounds like L-Theanine: while preclinical studies have showed that L-Theanine triggers neural and vascular activations (Yokogoshi et al., 1998; Kimura and Murata, 1971; Kakuda et al., 2002; Siamwala et al., 2013), such effects have not been conclusively demonstrated in humans (Dodd et al., 2015). Finally, enhancing reproducibility is essential in the research field. It entails identifying experimental conditions for reaching effective interventions. For polyphenol interventions, conditions like hypercapnia or hypoxia are suggested (Gratton et al., 2020; Decroix et al., 2018).

Advances in fNIRS methodology will be essential in democratizing its application in nutrition research. These advancements will enable researchers to conduct novel investigations and expand the scope of studies in the field. Technological innovations, particularly in hardware designed to mitigate motion artifacts, hold the potential to facilitate a seamless transition from traditional laboratory setups to real-world scenarios, using portable fNIRS devices (Huang et al., 2022). This transition would permit to scale up of studies and to test dietary effects in people’s daily lives as they occur (Pinti et al., 2018). Moreover, these advancements encompass a deeper understanding of the isolated mechanisms of nutritional components, facilitating the exploration of complex interventions involving various foods and beverages. Ultimately, researchers can enhance their understanding of the link between brain activity, nutrition, and behavior, gaining insights into the intricate relationships shaping overall brain health. As interest in the impact of nutrition on brain health and mental well-being grows, this knowledge sets the stage for precise and impactful nutritional interventions seamlessly integrated into daily life.
